# Multifunctional and Collaborative Protection of Proteins, Peptides, Phenolic Compounds, and Other Molecules against Oxidation in Apricot Seeds Extracts

**DOI:** 10.3390/antiox11122354

**Published:** 2022-11-28

**Authors:** María Concepción García, Víctor Lombardo-Cristina, María Luisa Marina

**Affiliations:** 1 Departamento de Química Analítica, Química Física e Ingeniería Química, Universidad de Alcalá, Ctra. Madrid-Barcelona Km. 33.600, 28871 Alcalá de Henares, Madrid, Spain; 2 Instituto de Investigación Química “Andrés M. del Río”, Universidad de Alcalá, Ctra. Madrid-Barcelona Km. 33.600, 28871 Alcalá de Henares, Madrid, Spain

**Keywords:** antioxidant activity, apricot seed, pressurized liquid extraction, proteins, phenolic compounds, mass spectrometry

## Abstract

Antioxidant activity studies usually focus on a single type of molecule and do not consider possible collaborations among different molecules. The purpose of this work was to obtain multicomponent extracts exerting protection against oxidation from apricot seeds and to study the individual role of these components in the whole protection. Pressurized liquid extraction was employed to obtain extracts, and a response surface methodology enabled exploration of the effect of extraction conditions on the composition and prevalence of the antioxidant mechanism. Extractions carried out at 170 °C, in up to 7% ethanol, and for up to 25 min guaranteed multifunctional protection against oxidation by the collaboration of different molecules. While phenolic compounds were the main contributors to radical-scavenging capacity (R^2^ = 90% for ABTS and 88% for DPPH), proteins and phenolic compounds showed similar roles in the whole reducing power (proteins (R^2^ = 86%) and TPC (R^2^ = 90%)), and other compounds inhibited the formation of hydroxyl radicals and, especially, the peroxidation of lipids. The presence of peptides modified the antioxidant protection of extracts. UHPLC-Q-Orbitrap-MS/MS confirmed the presence of phenolic compounds and other antioxidant molecules. The presence of different kinds of molecules led to a multifunctional and collaborative protection against oxidation that could not be exerted by individual molecules.

## 1. Introduction

Apricot seeds represent about 15% of the apricot (*Prunus armeniaca* L.). The apricot processing industry releases 300,000 tons of this waste per year. Nevertheless, apricot seeds have a high content of proteins (23–27%) and antioxidant molecules. Thus, the recovery of these valuable molecules within the apricot seed is of great interest [1,2,3,4,5].

Natural antioxidants molecules are greatly appreciated as an alternative to synthetic ones. Antioxidants prevent the oxidation of intracellular compounds by the removal of electrons or hydrogen atoms. This protection can be exerted by different mechanisms, with those antioxidants that play roles in multiple protection mechanisms or that show synergic effects being of great interest. The inhibition of hydroxyl radical formation is a very important mechanism for protecting cells from oxidative damage since hydroxyl radicals are highly reactive. Hydroxyl radicals are able to damage DNA and generate 8-hydroxyguanosine that is involved in cancer progression [6]. Scavenging of reactive oxygen species (ROS) is the mechanism followed by endogenous antioxidant enzymes (superoxide dismutase, glutathione peroxidase, peroxiredoxin, glutaredoxin, thioredoxin, and catalase) to neutralize or capture ROS [7]. During this process, the antioxidants become less reactive free radicals and are easily neutralized by other antioxidants. Moreover, antioxidants are also reducing agents that can deactivate metal ions such as Cu^2+^ or Fe^2+^. Transition metals react with hydrogen peroxide to produce hydroxyl radicals that promote oxidative damage. On the other hand, the oxidation of polyunsaturated fatty acids results in hydroperoxides that can react at cell level with proteins, nucleic acids, and glutathione, altering their normal functions. Lipid oxidation has been related with carcinogenesis, cardiovascular diseases, neurodegeneration, and aging [8]. Furthermore, lipid peroxidation results in undesirable volatiles in foods.

The extraction of value molecules from a waste material such as apricot seeds requires the use of sustainable conditions, since it would make no sense to employ polluting methodologies. An alternative to the nonsustainable methodologies usually employed to extract proteins and phenolic compounds is pressurized liquid extraction (PLE) [1]. PLE is a clean extraction technique that enables the reduction of extraction times and solvent consumption and results in a high yield. It uses green organic solvents submitted at high temperatures and pressures [9,10]. PLE has been used for the extraction of small molecules such as polyphenols [9,11]; however, it has never been applied for the extraction of phenolic compounds from apricot seeds. Moreover, recent publications have demonstrated the potential of PLE to extract large molecules such as proteins [12]; however, it has never been employed to extract proteins from apricot seeds.

Phenolic compounds and, to a lesser extent, proteins and peptides are the antioxidant molecules that are usual studied [9,11,12,13]. Nevertheless, most works focus just on single type of molecule (proteins, peptides, or phenolic compounds), and possible synergies among them or their individual contributions to the whole antioxidant activity of a sample have not been considered.

The aim of this work was to obtain extracts providing multifunctional protection against oxidation from apricot seeds using pressurized liquid extraction and to evaluate, for the first time, the individual contribution of proteins, peptides, phenolic compounds, and other molecules to the whole activity. Proteins, peptides, phenolic compounds, and other antioxidant molecules from apricot seeds showed different roles, and their simultaneous presence led to a unique protection based on different mechanisms that could not be observed in extracts containing individual molecules. These extracts could be of high industrial applicability.

## 2. Materials and Methods

### 2.1. Chemicals and Samples

All chemicals and reagents were of analytical grade. Water was obtained daily in a Milli-Q system from Millipore (Bedford, MA, USA). Ethanol (EtOH), methanol (MeOH), acetonitrile (ACN), urea, glycerol, and hydrochloric acid (HCl) were from Scharlau (Barcelona, Spain). Tris(hydroxymethyl)aminomethane (Tris), sodium dodecyl sulphate (SDS), sodium hydroxide (NaOH), and phosphate buffer (PB) were from Merck (Darmstadt, Germany). Albumin from bovine serum, dithiothreitol (DTT), o-phthaldialdehyde (OPA), L-glutatione (GSH), 2,2′-azino-bis(3-ethylbenzothiazoline-6-sulfonic acid) diammonium (ABTS), sodium tetraborate, sodium carbonate (Na_2_CO_3_), β-mercaptoethanol (β-ME), ethylenediaminetetraacetic acid (EDTA), acetic acid (AA), trifluoroacetic acid (TFA), potassium persulfate, 2,2-diphenyl-1-picrylhydrazyl (DPPH), 1,10-phenantroline, hydrogen peroxide (H_2_O_2_), pepsin, pancreatin, potassium ferricyanide (K_3_[Fe(CN)_6_]), iron (III) chloride (FeCl_3_), iron (II) chloride (FeCl_2_), ferrous sulphate, ammonium thiocyanate, 6-hydroxy-2,5,7,8-tetramethylchroman-2-carboxylic acid (Trolox), Folin–Ciocalteu reagent, gallic acid, trichloroacetic acid (TCA), linoleic acid, 3-[4,5-dimethylthiazol-2-yl]-2,5-diphenyltetrazolium bromide (MTT), dimethyl sulfoxide (DMSO), antibiotics (penicillin, streptomycin, and amphotericin), fetal bovine serum, and Dulbecco’s Modified Eagle’s Medium (DMEM) were purchased from Sigma-Aldrich (Saint Louis, MO, USA). Laemmli buffer, Tris/glycine/SDS running buffer, Mini-Protean Precast gels, and BioSafe Coomassie stain CBB G-250 were acquired at Bio-Rad (Hercules, CA, USA), while protein marker VI and thiourea were from PanReac AppliChem (Barcelona, Spain). OFF-GEL IPG buffer (pH 3–10) and OFF-GEL ampholytes (pH 3–10) buffer were from GE Healthcare (Uppsala, Sweden). Alcalase 2.4 L FG was kindly donated by Novozymes Spain S.A (Madrid, Spain). Super-absorbent powder was from Velp Scientifica (Monza y Brianza, Italy). Apricot seeds were from Green Planet Shop (Murcia, Spain).

### 2.2. Extraction

Seeds from apricot were ground in a domestic mill, dried at 103 ± 2 °C until constant weight, and stored at −20 °C until use. Extraction by PLE was performed with an accelerated solvent extractor system (ASE 150, Dionex, Sunnyvale, CA, USA). Solvents (water, EtOH, and mixtures) were degassed in an ultrasounds bath for 30 min. PLE was initially preheated for 6 min at 1500 psi and purged with nitrogen for 100 s. Extraction was carried out by mixing 1 g of apricot seeds with 7 g of sand. This mixture was placed into a 10 mL stainless steel extraction cell with a cellulose filter at the bottom to avoid suspended particles from leaking. All extracts were brought to a final volume of 25 mL and centrifuged at 4000× *g* for 10 min to collect supernatants.

An incomplete factorial experimental design, based on three levels, was employed to optimize the extraction temperature, the percentage of EtOH in the solvent, and the extraction time. The protein content, the total phenolic content (TPC), and the activity of extracts in five different antioxidant mechanisms were employed as response variables. A Box–Behnken experimental design with three levels and five central points was carried out. The experimental data were fitted to a quadratic model using the following second-order polynomial model equation for every response variable:

$$Y={\;\beta }_{0}+\sum^{\;}\beta_{i}X_{i}+\sum^{\;}\beta_{\mathrm{ii}}X_{i}{}^{2}+\sum^{\;}\beta_{\mathrm{ij}}X_{i}X_{j}$$

where Y is the response variable, β_0_ is the constant, β_i_ is the linear regression coefficient, β_ii_ is the quadratic regression coefficient, β_ij_ is the interaction regression coefficient, and X_i_ and X_j_ are independent variables. The determination coefficient (R^2^) and the analysis of variance (ANOVA), at a confidence level of 95%, were employed to evaluate the fitting of data to this mathematical model.

Proteins from apricot seeds were also extracted, for comparison purposes, using a nonsustainable method described in García et al. [13]. Briefly, it consisted of mixing ground seeds (0.5 g) with 100 mM Tris-HCl (pH 7.5) containing 0.5% SDS (w/v) and 0.5% DTT (w/v) using a high-intensity focused ultrasound probe (HIFU, model VCX130, Sonics Vibra-Cell, Hartforf, CT, USA) at 30% amplitude for 1 min, followed by centrifugation (10 min, 4000× *g*). Afterwards, supernatant was collected and proteins were precipitated with 10 mL of cold acetone at 4 °C for 15 min. After centrifugation (10 min, 4000× *g*), the pellet was retained.

### 2.3. Determination of Total Protein Content and Total Phenolic Content (TPC)

The protein content in extracts was determined by combustion following the Dumas method [14]. For that purpose, a Dumas equipment N702 from Velp Scientifica (Monza y Brianza, Italy) was employed. The instrument was calibrated with EDTA (9.48% N). Extracts were mixed with an absorbent (Super-Absorbent Powder) in the following ratio: 100 mg of absorbent component and 200 mg of extract (final volume, around 200 μL). Extracting solvents were used as blanks. The conversion factor was 6.25.

The Folin–Ciocalteu method was used for determining the TPC in extracts [15]. This method consists of mixing 20 μL of sample or standard (gallic acid, 0–100 mg/L), 200 μL of Folin–Ciocalteu reagent (previously diluted ten times), and 160 μL of 1 M Na_2_CO_3_. The mixture was performed in 96-well plates and incubated at room temperature (RT) for 30 min in the dark. Finally, the absorbance was measured at 750 nm in a plate reader (Multiskan Sky, Thermo Scientific, Waltham, MA, USA). The TPC was expressed as milligrams of gallic acid equivalents (GAE)/100 mg sample.

### 2.4. Protein Hydrolysis and Determination of Peptide Content

Proteins in extracts were hydrolyzed through gastrointestinal digestion or with Alcalase enzyme. Digestions were carried out in a Thermomixer (Eppendorf, Hamburg, Germany). Simulated gastrointestinal digestion consisted of a first digestion with pepsin followed by digestion with pancreatin enzymatic mixture. Pepsin digestion was performed by mixing the extract with the enzyme at a 1:35 enzyme:substrate ratio and adjusting the pH to 2.0 with 1 M HCl. The mixture was incubated at 37 °C for 1 h. Next, pancreatin digestion was performed by mixing the extract with the enzyme at a 1:25 enzyme:subtrate ratio and adjusting the pH to 7.0–8.0 with 1 M NaOH. This digestion step was also carried out at 37 °C for 2 h.

Digestion with Alcalase enzyme was carried out by mixing 0.15 UA/g protein, adjusting the pH of extracts to 6.5–8.5, and incubating the mixture for 4 h at 50 °C and 750 rpm. In all cases, reactions were stopped by increasing the temperature to 100 °C for 10 min and centrifuging at 7000× *g* for 10 min. Supernatants, containing peptides, were collected.

The peptide content in hydrolysates was determined by the OPA assay [16]. The OPA reagent (9 mL) was prepared by mixing 4.5 mL of 100 mM sodium tetraborate, 1.8 mL of 5% SDS (w/v), 18 μL of β-ME, 180 μL of 40 mg/mL OPA in MeOH, and 2.5 mL of water. OPA reagent (100 μL) was mixed with 2.5 μL of sample in 96-well plates and left to stand for 8 min at RT. Afterwards, the absorbance was measured at 340 nm. A calibration curve was prepared using GSH (0–2 mg/mL) as peptide standard, and the peptide concentration of extracts was determined by interpolation in that calibration curve.

### 2.5. In Vitro Evaluation of the Antioxidant Capacity of Extracts and Hydrolysates

The capacity of extracts and hydrolysates to scavenge free radicals (ABTS and DPPH), to avoid the formation of hydroxyl radicals, to reduce Fe (III), and to inhibit lipids peroxidation was determined using the assays described below. Blanks, in every case, were obtained by using extracting solvents. Three replicates were averaged in every case.

#### 2.5.1. Ability to Scavenge ABTS Radicals

The capacity of extracts to scavenge ABTS^·+^ was evaluated following the method described by González-García et al. [17], with some modifications. A stock solution of ABTS^·+^ was prepared by mixing 7 mM ABTS and 2.6 mM potassium persulphate in 10 mM PB (pH 7.4) and keeping this in the dark for 12–16 h. The ABTS^·+^ working solution was next prepared by adding 10 mM PB (pH 7.4) to the stock solution until the absorbance, at a wavelength of 734 nm, reached the value of 0.70 ± 0.02. Samples (1 μL) were mixed in 96-well plates with 100 μL of ABTS^·+^ working solution for 6 min at RT. The resulting absorbance was next measured at 734 nm, and the capacity was calculated by the following equation:

$$\mathrm{ABTS}\;\mathrm{radical}\;\mathrm{scavenging}\;\mathrm{capacity}\;\left(\%\right)=\left(\frac{{\mathrm{Abs}}_{\mathrm{blank}\;}{-\;\mathrm{Abs}}_{\mathrm{sample}}}{{\mathrm{Abs}}_{\mathrm{blank}}}\right)\;\times \;100$$

where Abs_blank_ is the absorbance corresponding to the solvent with ABTS^+^ and Abs_sample_ is the absorbance corresponding to the sample with ABTS^+^. GSH (0.5 mg/mL) was used as positive control.

#### 2.5.2. Ability to Scavenge DPPH Radicals

DPPH radical-scavenging capacity was evaluated following the method described by Bobo-García et al. [18]. Samples (20 μL) were mixed in 96-well plates with 180 μL of 0.1 mM DPPH solution in 95% EtOH (v/v). The mixture was kept in the dark at RT for 30 min, and afterwards, the absorbance was measured at a wavelength of 517 nm. The DPPH radical-scavenging capacity was calculated with the following equation:

$$\mathrm{DPPH}\;\mathrm{radical}\;\mathrm{scavenging}\;\mathrm{capacity}\;\left(\%\right)=\left(1\;-\;\frac{{\mathrm{Abs}}_{\mathrm{sample}\;}{-\;\mathrm{Abs}}_{\mathrm{control}}}{{\mathrm{Abs}}_{\mathrm{blank}}}\right)\;\times \;100$$

where Abs_sample_ is the absorbance of the sample with DPPH, Abs_control_ is the absorbance of the sample with 95% EtOH, and Abs_blank_ is the absorbance of the solvent with DPPH. Trolox (0.05 mg/mL) was used as positive control.

#### 2.5.3. Ability to Avoid Hydroxyl Radical Formation

This assay was carried out following the method described by Hernández-Corroto et al. [16]. Hydroxyl radicals were formed from H_2_O_2_ that oxidizes Fe (II) to Fe (III) through Fenton reaction. For that purpose, 50 μL of 3 mM 1,10-phenanthroline in 100 mM PB (pH 7.4) was mixed with 50 μL of 3 mM ferrous sulphate, 50 μL of sample, and 50 μL of 0.01% H_2_O_2_ (v/v) in 96-well plates. The mixture was incubated at 37 °C for 1 h, with continuous agitation. The ability to inhibit the formation of hydroxyl radicals was evaluated by monitoring the absorbance corresponding to the complex Fe (II)-phenanthroline at a wavelength of 536 nm and calculated by the following equation:

$$\mathrm{Hydroxyl}\;\mathrm{radical}\;\mathrm{formation}\;\mathrm{inhibition}\;\left(\%\right)=\left(\frac{{\mathrm{Abs}}_{\mathrm{sample}}{\;-\;\mathrm{Abs}}_{\mathrm{blank}}}{{\mathrm{Abs}}_{\mathrm{control}\;}{-\;\mathrm{Abs}}_{\mathrm{blank}}}\right)\;\times \;100$$

where Abs_sample_ is the absorbance of the sample, Abs_blank_ is the absorbance of the solvent, and Abs_control_ is the absorbance of a control solution containing water instead of H_2_O_2_. GSH (1 mg/mL) was used as positive control.

#### 2.5.4. Ferric Reducing Antioxidant Power (FRAP)

The reducing power was evaluated by following the method developed by Gomaa [19], with some modifications. For that purpose, the sample (25 μL) was mixed with 25 μL of 0.2 M PB (pH 6.6) and 50 μL of 1% K_3_[Fe(CN)_6_] (w/v) and incubated in the Thermomixer for 20 min at 50 °C and 750 rpm. This reaction was stopped by adding 50 μL of 10% TCA (w/v). The increase in absorbance at 700 nm occurring when Fe (III), from the ferricyanide complex, was reduced to Fe (II) by antioxidant compounds was next measured by mixing 100 μL of the mixture with 600 μL of 0.08% FeCl_3_ (w/v) and standing this for 3 min. The reducing capacity was calculated by the following equation:

$$\mathrm{Reducing}\;\mathrm{power}=\frac{{\mathrm{Abs}}_{\mathrm{sample}}}{\mathrm{peptide}\;\mathrm{content}}$$

where Abs_sample_ is the measured absorbance and peptide content is that obtained by OPA assay. The percentage of inhibition related to the reduction power signal corresponding to a solution of 1 mg/mL GSH was calculated.

#### 2.5.5. Ability to Inhibit the Peroxidation of Lipids

The capacity of extracts and hydrolysates to inhibit the oxidation of lipids was monitored by the method described by Hernández-Corroto et al. [16]. The sample (20 μL) was mixed with 20 μL of 1.3% linoleic acid (v/v) in EtOH and 10 μL of water and let stand for 144 h at 40 °C in the dark. Afterwards, the degree of oxidation of the linoleic acid was evaluated by mixing 2.5 μL of this solution with 175 μL of 75% EtOH (v/v), 2.5 μL of 30% ammonium thiocyanate (w/v), and 2.5 μL of 20 mM FeCl_2_ in 3.5% HCl (v/v). After standing for 6 min at RT, the absorbance corresponding to ferric thiocyanate was measured at 500 nm. The following equation was employed to determine the capacity of inhibition of lipid peroxidation:

$$\mathrm{Lipids}\;\mathrm{peroxidation}\;\mathrm{inhibition}\;\mathrm{capacity}\;\left(\%\right)=\left(1\;-\;\frac{{\mathrm{Abs}}_{\mathrm{sample},t144}{\;-\;\mathrm{Abs}}_{\mathrm{sample},t0}}{{\mathrm{Abs}}_{\mathrm{blank},t144\;}{-\;\mathrm{Abs}}_{\mathrm{blank},t0}}\right)\;\times \;100$$

where Abs_sample,t144_ and Abs_blank,t144_ are the absorbances of the sample and the solvent, respectively, after 144 h incubation; Abs_sample,t0_ and Abs_blank,t0_ are the initial absorbances of the sample and the solvent, respectively.

### 2.6. Antiproliferative Effect of Extracts

Antiproliferative studies were carried out with HeLa (cervical cancer) cells from the American Type Culture Collection ATCC (Rockwell, MD, USA). HeLa cells were cultured in DMEM supplemented with penicillin (100 U/mL), streptomycin (100 μg/mL), amphotericin (250 ng/mL), and 10% fetal bovine serum. Cells were kept at 37 °C in a humidified atmosphere with 5% CO_2_, until use.

Before the assay, cells were cultured for three days in 24-well plates (density was 8000 cells/well) and incubated with 50 μL of extracts for 24 h at 37 °C with 5% CO_2_. After that, 50 μL of 5 mg/mL MTT stock solution in PB was added to each well and this was incubated for 3–4 h. Then, the culture medium was removed and the formed blue formazan crystals were dissolved in 500 μL DMSO. The absorbance was measured at a wavelength of 570 nm with background subtraction at 630–690 nm. The percentage of cell viability was calculated by the following equation:

$$\mathrm{Cell}\;\mathrm{viability}=\frac{{\mathrm{Abs}}_{\mathrm{sample}}}{{\mathrm{Abs}}_{\mathrm{control}}}$$

where Abs_sample_ is the absorbance of remaining blue formazan after being treated with extracts and Abs_control_ is the absorbance obtained when adding the employed solvent.

### 2.7. Characterization of Proteins and Phenolic Compounds

#### 2.7.1. Separation of Proteins by SDS–Polyacrylamide Gel Electrophoresis (SDS-PAGE)

Proteins were separated by SDS-PAGE using a Bio-Rad Mini-Protean system (Hercules, CA, USA). Samples were dissolved in Laemmli buffer (62.5 mM Tris-HCl (pH 6.8), 25% glycerol (v/v), 2% SDS (w/v), 0.01% bromophenol blue, and 5% β-ME (v/v)) at a 1:1 ratio and heated at 100 °C for 5 min. Gels were loaded with 20 μL of samples or 8 μL of the protein standard ladder and then immersed in a cuvette containing the running buffer (25 mM Tris-HCl (pH 8.3), 192 mM glycine, and 0.1% SDS (w/v)). Separation was carried out by applying 70 V for 5 min and 150 V for 1 h. Proteins were next fixed with a mixture of 40% MeOH (v/v) and 10% AA (v/v) by shaking for 30 min. After removal of that mixture, proteins were stained with BioSafe Coomassie solution for 1 h. Finally, gels were washed three times with Mili-Q water.

#### 2.7.2. Separation of Proteins by OFF-GEL Isoelectrofocusing (IEF)

Proteins were fractionated in a 24-well set up by IEF using the Agilent 3100 OFF-GEL fractionator (Agilent Technologies, Forest Hill, VIC, Australia) and pH gradient (IPG) gel strips (General Electric Healthcare, Freiburg, Germany) from pH 3 to 10. The 1.25 X protein OFF-GEL stock solution (15 mL) was prepared by mixing 7.56 g urea, 180 mg DTT, 2.73 g thiourea, 1.8 mL glycerol (12% (v/v)), 180 μL OFF-GEL ampholytes (pH 3–10) buffer, and Milli-Q water. Prior to the separation, gels and electrodes pads were hydrated with 40 μL of a mixture (1:4) of water:1.25 X stock solution. Next, hydrated IPG strip gels were loaded with 150 μL of extracts, previously diluted at a 1:4 ratio with the stock solution. Separation of proteins was carried out at 64 kV/h, 50 μA, and 200 mW while the voltage was varied as follows: 300 V for 2 h, 600 V for 2 h, 1500 V overnight, and finally, 4500 V. Electrode pads were replaced by new pads rehydrated after every 24 h of operation. Recovered fractions were then separated by reversed-phase high-performance liquid chromatography (RP-HPLC) in an Agilent Technologies 1100 series HPLC (Palo Alto, CA, USA) using an Aeris Widepore XB C18 (100 nm × 2.1 mm ID, 3.6 μm particle size) column from Phenomenex (Torrance, CA, USA). Chromatographic conditions were: elution gradient, 5–60% B for 20 min, 60–95% B for 3 min, and 95% B for 2 min; mobile phases, water + 0.1% TFA (v/v) (mobile phase A) and ACN + 0.1% TFA (v/v) (mobile phase B); flow-rate, 0.3 mL/min; injection volume, 10 μL; temperature, 25 °C; fluorescence detection, λ_exc_ of 280 nm and λ_emi_ of 360 nm.

### 2.8. Identification of Phenolic Compounds

The identification of phenolic compounds in extracts was carried out by ultraperformance HPLC (UPLC)–Quadrupole (Q)–Orbitrap tandem mass spectrometry (MS/MS). UHPLC Dionex Ultimate 3000, with degasser, binary pump, autosampler, and column compartment, was coupled to a Q Exactive Orbitrap MS system with an electrospray ionization source, all from Thermo Scientific (Waltham, MA, USA). Reversed-phase separation was performed on an Ascentis Express ES-C18 column (100 mm × 2.1 mm I.D., 2.7 μm particle size) with an Ascentis Express guard column (5 mm × 2.1 mm I.D., 2.7 μm particle size), both from Supelco (Bellefonte, PA, USA). Chromatographic conditions were: elution gradient, 3% B for 5 min, 3–20% B for 35 min, and 20–95% for 3 min; mobile phases, water + 0.3% AA (v/v) (mobile phase A) and ACN + 0.3% AA (v/v) (mobile phase B); flow rate, 0.3 mL/min; injection volume, 10 μL; temperature, 25 °C.

The mass spectrometer (MS) was operated in the negative-ion mode. Key parameters were: spray voltage, −3.0 kV; sheath gas flow rate, 60 AU; auxiliary gas flow rate, 30 AU; sweep gas flow rate, 0 AU; capillary temperature, 280 °C; auxiliary-gas-heater temperature, 350 °C. The MS was operated in the scan mode with a resolution of 70,000 FWHM (at m/z 200) and in data-dependent MS/MS with a resolution of 17,500 FWHM. The stepped normalized collision energy was 35 eV, and the scan range was m/z 113.4–1700. Data acquisition and processing were carried out with the Compound Discoverer 3.2 software (Thermo Scientific, Waltham, MA, USA). Identification was performed by comparing mass and retention time of precursor ions and their fragmentation patterns using ChemSpider, mzCloud, mzVault, FooDB, and Mass Bank libraries. Only compounds that appeared in two separate samples, at least, by duplicate, were accepted.

### 2.9. Statistical Analysis

Experimental design and statistical analysis were performed using Statgraphics Centurion XVII software (Statpoint Technologies, Inc., Warranton, VA, USA). Values are expressed as mean ± standard deviation. The analysis of variance (ANOVA) was performed using a significant level of 0.05.

## 3. Results and Discussion

### 3.1. Optimization of Extraction Parameters Using Pressurized Liquids to Obtain Extracts with Multiple and High Protection against Oxidation

A Box–Behnken experimental design was employed to find out the optimal conditions in pressurized liquid extraction to obtain extracts with a high protection against oxidation through different antioxidant mechanisms: scavenge of free radicals (both ABTS and DPPH radicals), inhibition of hydroxyl radical formation, reduction of oxidizing compounds, and inhibition of lipids peroxidation. Three extraction parameters were evaluated: temperature (50 to 170 °C), time (5 to 25 min), and solvent composition (water–EtOH, 0–100%). The experimental design established the 17 experiments, included in Table 1, and Figure S1 shows the images of all extracts. Twelve points corresponded to the factorial design, while five were control points (experiments 1, 5, 9, 10, and 15). The color of extracts varied from transparent to brownish, with darker colors observed in extracts obtained at 170 °C. Color development was probably due to the Maillard reaction.

**Figure 1:**
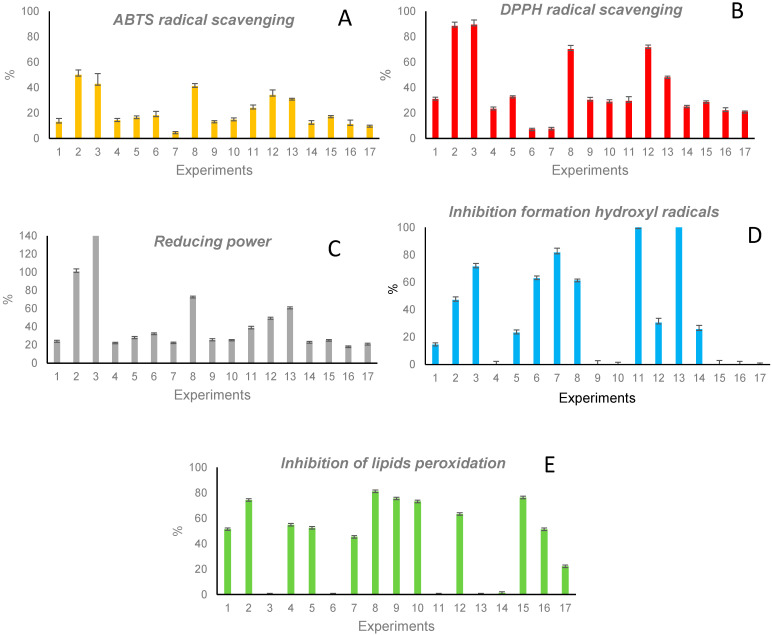
Capacity to scavenge ABTS (**A**) and DPPH (**B**) radicals, to reduce oxidant compounds (**C**), to inhibit the formation of hydroxyl radicals (**D**), and to inhibit lipids peroxidation (**E**) of the 17 extracts obtained by PLE.

Results corresponding to the antioxidant protection of the 17 extracts are included in Figure 1A–E. The highest radical-scavenging capacity, for both ABTS and DPPH radicals, was observed in extracts 2, 3, 8, and 12 (those obtained at a temperature of 170 °C), while extract 7 (obtained at 40 °C) showed a very low capacity in both cases (see Figure 1A,B). A similar activity profile was observed for the reducing power (Figure 1C, results related to a control solution of 1 mg/mL GSH). The highest inhibition in the formation of hydroxyl radicals was observed in extracts 3, 7, 11, and 13, and the lowest in extracts 4, 9, 10, and 15–17 (all obtained at 50–100% EtOH and 40–105 °C) (see Figure 1D). The capacity to avoid lipids peroxidation (Figure 1E) was high in extracts 2, 8, 9, 10, 12, and 15 (obtained at 50–100% EtOH and 105–170 °C), and almost no capacity was observed in extracts obtained with 0% EtOH (3, 6, 11, and 13).

### 3.2. Contribution of Proteins and Phenolic Compounds to the Protection against Oxidation Observed in the Extracts Obtained by PLE

The protein content and total phenolic content (TPC) in the 17 extracts established by the Box–Behnken experimental design were determined and are included in Table 1. The highest protein content was observed in extract 3 (22 ± 1 mg/100 mg apricot seed), obtained at 0% EtOH, at the highest temperature (170 °C), and with an extraction time of 15 min. This content is four times the protein content obtained using a conventional and nonsustainable procedure [13] (5.4 ± 0.3 mg of proteins/100 mg apricot seeds) and demonstrates the potential of PLE as an alternative to the conventional and nonsustainable methods usually employed for the extraction of proteins. The lowest protein yield was observed in extracts 17 and 16 (8.8 ± 0.3 and 9 ± 1 mg/100 mg apricot seed, respectively), obtained at higher percentages of EtOH (50–100%), medium–high temperatures, and with 5 min extraction.

A higher variation in TPC was observed within extracts. The highest value corresponded to extracts 3 and 2 (0.63 ± 0.04 and 0.62 ± 0.01 mg GAE/100 mg seeds, respectively), obtained at 0–50% EtOH and at a high temperature, while the lowest TPC was in extracts 16 and 4 (0.00 ± 0.01 mg GAE/100 mg seeds), obtained at 100% EtOH and 105 °C. The highest yield (0.63 mg GAE/100 mg seeds) was much higher than that obtained by other authors in apricot seeds using conventional extraction with acetone (0.209 mg GAE/100 mg seeds) [2], water, EtOH or MeOH (0.33 mg GAE/100 mg seeds) [19], and MeOH or acetone (0.16 mg GAE/100 mg seeds) [20].

From these results, extract 3 showed simultaneously a high amount of proteins as well as TPC, suggesting that the lowest EtOH percentage and the highest extraction temperature could promote the extraction of both proteins and phenolic compounds. Additionally, a comparison of contents in experiment 3 with the corresponding contents in experiment 2, obtained at a higher EtOH percentage (50%) and at the same temperature (170 °C), resulted in a lower protein extraction while keeping TPC. A higher increase in the percentage of EtOH, keeping the temperature at 170 °C, (experiment 12, 100% EtOH) reduced the extraction of both proteins and TPC. This result is consistent with previous works [21] demonstrating the low extractability of phenolic compounds at very high EtOH percentages.

The comparison of protein contents and TPC in extracts, with results shown in Figure 1, enabled interesting information on the contribution of proteins and phenolic compounds to every antioxidant mechanism to be observed. Extracts showing the highest capacity to scavenge ABTS and DPPH radicals (extracts 2, 3, 8, and 12) also showed the highest TPC values. The high correlation of TPC with these antioxidant mechanisms (R^2^ = 90% for ABTS and 88% for DPPH) revealed the significant role of phenolic compounds. Additionally, proteins could play a secondary role in these mechanisms. Despite the reducing power profile being similar to the scavenging capacity, this activity was highly correlated with both the protein content (R^2^ = 86%) and TPC (R^2^ = 90%) and not only with TPC, as in the case of the scavenging capacity. This result suggested that the contribution of proteins to the reducing power was higher than their contribution to the radical- scavenging mechanism.

The extracts that highly inhibited the formation of hydroxyl radicals (3, 7, 11, and 13) showed a slight correlation with the protein content and TPC, and it is likely that other antioxidant compounds, in addition to proteins and phenolic compounds, could be contributing to this activity. Unlike previous mechanisms, the inhibition of lipid peroxidation was not correlated with the protein content or TPC, and it was likely due to the contribution of other antioxidant compounds different to proteins or phenolic compounds.

### 3.3. Determination of Most Suitable PLE Conditions to Obtain the Highest Content in Proteins and Phenolic Compounds and the Highest Protection against Oxidation

The results included in Table 1 and Figure 1 were employed to obtain mathematical models enabling the prediction of the optimal PLE conditions to extract proteins and phenolic compounds and to obtain extracts with a high capacity to avoid oxidative damage. For this purpose, the explanatory variables were the percentage of EtOH, the extraction time, and the extraction temperature, while the protein content, TPC, capacity to scavenge ABTS and DPPH radicals, ability to inhibit the formation of hydroxyl radicals, reducing power, and capacity to inhibit lipid peroxidation were the response variables. The mathematical models best predicting response variables were:

Protein content (mg/100 mg) = 12.68 − 0.04 X_1_ − 0.08 X_2_ + 0.20 X_3_ − 0.0002 X_1_ X_2_ + 0.0006 X_2_^2^ − 0.007 X_3_^2^

TPC (mg GAE/100 mg) = 0.42 − 0.0002 X_1_ − 0.008 X_2_ − 0.005 X_3_ − 0.000008 X_1_^2^ − 0.000009 X_1_ X_2_ + 0.00005 X_2_^2^ + 0.00008 X_2_ X_3_

ABTS scavenging capacity (%) = 35.66 − 0.27 X_1_ − 0.47 X_2_ + 0.26 X_3_ + 0.001 X_1_ X_2_ + 0.003 X_2_^2^

DPPH scavenging capacity (%) = 46.92 − 0.01 X_1_ − 1.1 X_2_ + 2.5 X_3_ − 0.001 X_1_^2^ + 0.002 X_1_ X_2_ − 0.01 X_1_ X_3_ + 0.006 X_2_^2^ + 0.005 X_2_ X_3_ − 0.06 X_3_^2^

Inhibition of hydroxyl radicals (%) = 104.34 − 2.09 X_1_ − 1.23 X_2_ + 3.05 X_3_ + 0.01 X_1_^2^ + 0.007 X_2_^2^ − 0.02 X_2_ X_3_

Reducing power (%) = 68.43 − 0.29 X_1_ − 1.2 X_2_ + 1.3 X_3_ + 0.003 X_1_^2^ − 0.004 X_1_ X_2_ + 0.008 X_2_^2^ + 0.01 X_2_ X_3_ − 0.06 X_3_^2^

Inhibition of lipid peroxidation (%) = − 65.28 + 1.91 X_1_ + 0.62 X_2_ + 2.74 X_3_ − 0.01 X_1_^2^ + 0.001 X_1_ X_2_ − 0.0003 X_1_ X_3_ − 0.002 X_2_^2^ + 0.005 X_2_ X_3_ − 0.12 X_3_^2^

where X_1_ is the percentage of EtOH, X_2_ is the extraction temperature, and X_3_ is the extraction time. Variables included in the mathematical models were those that were significant and those whose removal negatively affected R^2^ or resulted in a lack of validation. The mathematical models explained 90% of the protein content variability, 98% of the TPC variability, 94% of the variability in the capacity to scavenge ABTS and DPPH radicals, 73% of the variability in the capacity to inhibit the formation of hydroxyl radicals, 92% of the reducing power variability, and 89% of the variability in the capacity to inhibit lipid peroxidation.

**Figure 2:**
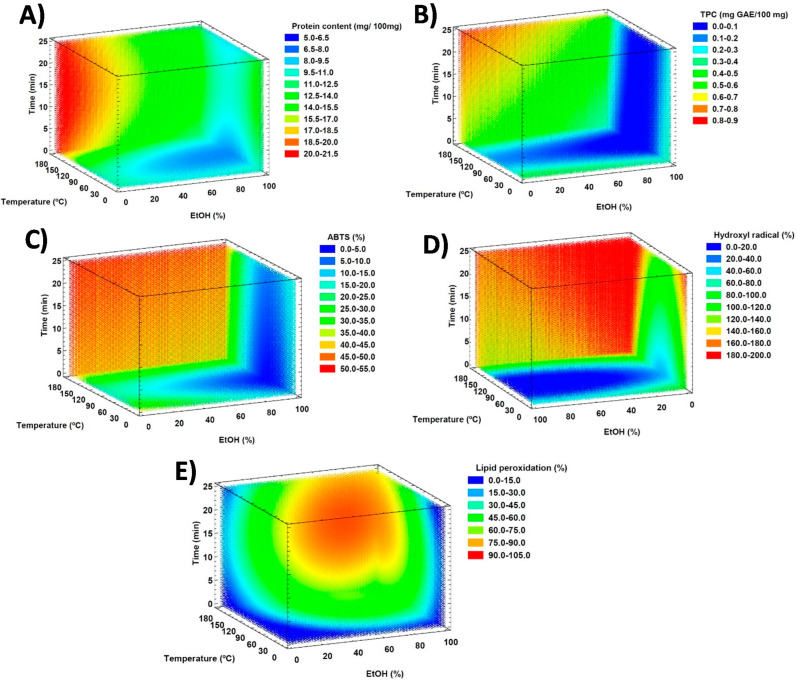
3D contour plots illustrating the effect of the extraction time, the extraction temperature, and the percentage of EtOH in the extraction yield of proteins (**A**), in the TPC (**B**), and in the capacity to scavenge free radicals (**C**), to avoid the formation of hydroxyl radicals (**D**), and to inhibit lipid peroxidation (**E**).

All mathematical models were validated by ANOVA (*p*-value > 0.05) except those predicting the capacity to scavenge DPPH radicals and the reduction power (FRAP). The 3D contour graphs showing the effect of the explanatory variables on the protein content, the TPC, the ABTS radical-scavenging capacity, the inhibition of the formation of hydroxyl radicals, and the capacity to avoid lipid peroxidation are included in Figure 2. 3D plots corresponding to the protein content (Figure 2A), the TPC (Figure 2B), the ABTS radical-scavenging capacity (Figure 2C), and the hydroxyl radical inhibition (Figure 2D) show some similarities. Indeed, all present increased values at high temperatures and low EtOH percentages, which confirms the contribution, in higher or lesser extent, of proteins and phenolic compounds to these antioxidant mechanisms. The capacity to avoid lipid peroxidation shows a different 3D plot with a higher yield at medium–high extraction times, temperatures, and percentages of EtOH.

The optimal conditions to obtain the highest protein content, TPC, and capacity to avoid oxidative damage by the different antioxidant mechanisms are grouped in Table 2 along with the protein content and TPC at these conditions. As stated previously, high temperatures promoted the extraction of proteins and phenolic compounds. Moreover, the highest antioxidant activity by the different mechanisms also required the use of high temperatures. Protein content in the extracts ranged from 19 to 21 mg/100 mg apricot seeds, while wider differences were observed among TPCs. The lowest TPC was obtained under the conditions for the highest capacity to inhibit hydroxyl radical formation (0.45 mg GAE/100 mg apricot seeds), probably due to the short extraction time (just 5 min). The highest TPC was observed in the extracts yielding the highest reducing power and inhibition of lipid peroxidation (0.75–0.72 mg GAE/100 mg apricot seed). It is possible that in these cases, other antioxidant compounds were being extracted, which would contribute to the highest TPC since this assay really evaluates the antioxidant capacity of any antioxidant compound.

Taking into account all these results, the use of high temperatures, up to 7% EtOH, and up to 25 min extraction times guarantees extracts with a multifunctional protection against oxidation from apricot seeds.

### 3.4. Contribution of Peptides Released by Gastrointestinal Digestion or Processing with a Food Enzyme to the Antioxidant Activity of Extracts

The exploitation of apricot seeds as a source of antioxidant compounds requires an evaluation of the effect of industrial processing and gastrointestinal digestion on this capacity since they can result in the modification of antioxidant molecules. Moreover, enzymatic digestion of proteins results in the release of peptides that could also contribute to the antioxidant activity. Therefore, the 17 extracts obtained under the conditions described in Table 1 were submitted to a simulated gastrointestinal digestion and to hydrolysis with Alcalase enzyme, and the peptide content and antioxidant properties of hydrolysates were evaluated. Table 3 shows the peptide content determined in the original extracts and after gastrointestinal digestion or processing with Alcalase (in all cases, an initial protein concentration of 2.4 mg/mL was used). The peptide content in the initial extracts ranged from 0.2 to 4.2 mg/mL, with the highest values observed in extracts 13, 3, and 11. After a simulated gastrointestinal digestion, the peptide content increased in most extracts, with the maximum value observed in extract 13 (5.3 mg/mL), followed by extracts 3 and 11. Nevertheless, the highest digestibility (higher increase in peptides related to the initial value) was observed in proteins present in extracts 2, 3, 4, and 8. In general, a lower peptide release was obtained when hydrolyzing with Alcalase. In this case, the highest digestibility was shown in extracts 3 and 4 and the highest peptide content was observed again in extract 13 (7.4 mg/mL). Regarding polyphenols, it was observed that the gastrointestinal or Alcalase digestion of extracts did not affect TPC values.

**Figure 3:**
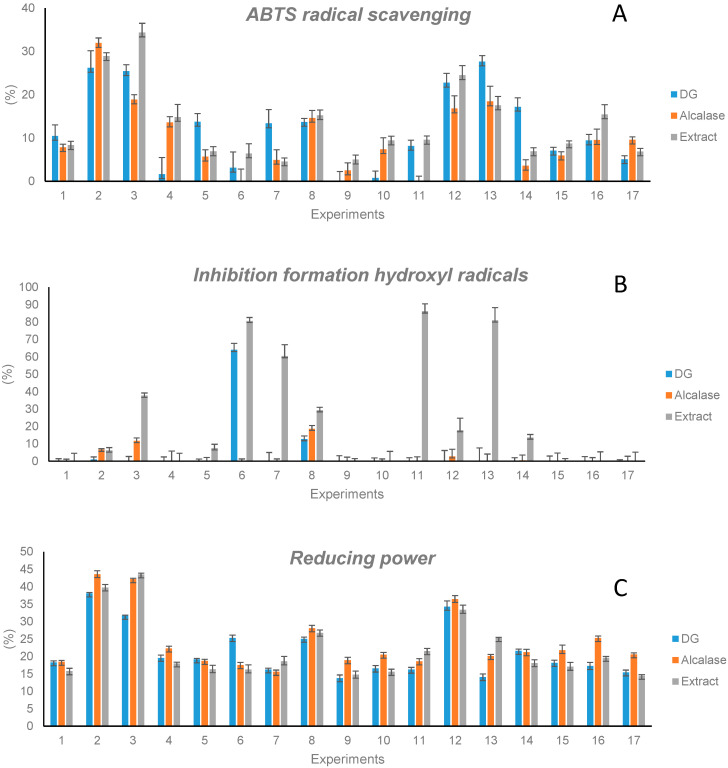
Capacity to scavenge ABTS radicals (**A**), to inhibit hydroxyl radicals (**B**), and to reduce oxidant molecules (**C**) of the 17 extracts and their hydrolyzates obtained by gastrointestinal digestion (GD) or by Alcalase hydrolysis.

In order to evaluate whether the release of peptides affected the antioxidant properties of extracts, the capacity of hydrolysates to scavenge ABTS radicals, to inhibit hydroxyl radical formation, and to reduce oxidant molecules was determined. Figure 3 compares the values obtained with hydrolysates with those of the original extracts at the same concentration (2.4 mg/mL). In general, all hydrolysates obtained by both GD and Alcalase processing showed a capacity to scavenge ABTS radicals. This capacity reached 28% in the case of the GD and 32% in the case of the Alcalase digestion. Nevertheless, a general behavior was not observed when comparing this to the activity of intact extracts. Indeed, the digestion of proteins did not affect the ABTS-scavenging capacity in some cases (extracts 1, 2, 8, 12, and 15), while in others, it resulted in a decrease (extracts 3, 4, 6, 9, 10, 11, and 16) or in an increase (extracts 5, 7, 13, 14, and 17).

The capacity to inhibit the formation of hydroxyl radicals decreased significantly when extracts were submitted to GD or Alcalase hydrolysis. This result demonstrated the contribution of proteins to this activity. On the other hand, the reduction capacity of extracts, in general, was retained when they were submitted to GD or to Alcalase hydrolysis. This capacity ranged from 13.8% to 38.1% in hydrolysates obtained by GD and from 18.5% to 43.6% when hydrolysates were obtained by digestion with Alcalase.

All these results support the combined contribution of proteins, peptides, and phenolic compounds to the capacity of extracts to scavenge free radicals and to the reducing power and the higher contribution of proteins, in comparison to phenolic compounds and peptides, to the inhibition of hydroxyl radical formation, which was significantly reduced when proteins were hydrolyzed.

### 3.5. Characterization of Extracts

The characterization of extracts was carried out by the study of their toxicity, by characterizing proteins, and by the identification of phenolics compounds and other compounds in extracts. Figure S2 shows the effect of the extracts on the proliferation of HeLa cancer cells. In general, the 17 extracts did not show cytotoxicity and were, therefore, suitable for their use in the food or pharmaceutical industries. This is a very important feature since it demonstrates that the cyanogenic compound amygdalin, present in apricot seeds [13], has not been extracted under the extraction conditions employed.

Proteins in extracts obtained under conditions grouped in Table 2 were characterized by the estimation of their molecular weights, through SDS-PAGE separation (Figure S3A), and their solubility, through isoelectrofocusing separation (Figure S3B). Extracts obtained under optimal conditions for the highest protein content, TPC, scavenging of ABTS and DPPH radicals, inhibition of hydroxyl radical formation, and reduction power yielded seven bands with the following molecular weights: one band at 75–100 kDa, one band close to 60 kDa, three bands between 48 and 30 kDa, one band at 20–25 kDa, and a last band between 17 and 11 kDa. The extract obtained under optimal conditions for lipid peroxidation inhibition showed a different profile, yielding only diffused bands at molecular weights below 11 kDa. This is probably due to the different extraction conditions employed to obtain this extract (76.4% EtOH).

Figure S3B shows the solubility of proteins extracted under conditions yielding the highest protein content (0% EtOH) at different pHs. Proteins showed minimum solubility at pH 4.9–5.0, 5.9–6.0, and 7.0–7.9. These pH ranges probably correspond to the isoelectric points of extracted proteins.

Extracted phenolic compounds were identified by UHPLC-Q-Orbitrap-MS/MS. Table 4 shows the phenolic compounds identified in the extracts obtained under conditions shown to have the highest TPC (TPC extract, obtained with 1.1% EtOH) and the highest inhibition of lipid peroxidation (ILP extract, obtained with 76.4% EtOH). These extracts were chosen because they were obtained under very different EtOH percentages. Eighteen different phenolic compounds were identified within the two extracts. Fifteen phenolic compounds were identified in the TPC extract, and fourteen in the ILP extract. Eleven phenolic compounds were common to both extracts, while others were observed just in the TPC extract (epigallocatechin, caffeic acid, 3,4-dihydrohyphenylpropionic acid, and DL-4-hydroxyphenyllactic acid) or in the ILP extract (phloretin, quercetin, and ethyl cahheate). Neochlorogenic and chlorogenic acids, catechin, epicatechin, and ferulic acid were previously identified in extracts from apricot seeds obtained with ethyl acetate and etanol [22], while chlorogenic acid derivatives were also observed in extracts obtained with EtOH [23].

Additionally, other compounds (see Table S1) were observed in TPC and ILP extracts. Within them, there were amino acids, nucleotides, metabolites, etc. Kynurenic acid has been found in cannabis and, along with tryptophan, has been related with neuroprotector functions [24]. Azelaic acid is well known by its antioxidant, antibacterial, and anti-inflammatory properties [25]. This acid, along with sebaic acid, citric acid, and α,α-trehalose, is used for skin care applications. Uridine is a nucleoside employed in the preparation of anti-inflammatory, antimicrobial, antiviral, and anticancer agents [26]. Some of these compounds were likely contributing to the antioxidant activity in extracts, especially in the ILP extract where a clear contribution of proteins and phenolics compounds was not observed (see Figure 1E). Finally, Table S2 shows other compounds present in both extracts that could not be identified. Interestingly, amygdalin was not identified in any extract, which confirmed that the selected conditions did not extract this toxic compound present in apricot seeds [13].

## 4. Conclusions

It has been possible to obtain an extract from apricot seeds with multifunctional protection against oxidation by the collaboration of different kinds of molecules. Pressurized liquid extraction enabled to tune the composition and, thus, the mechanisms of protection exerted by extracts to be modeled, since proteins, peptides, phenolic compounds, and other antioxidant molecules showed different contributions to the whole protection. While phenolic compounds were the main contributors to the scavenging of free radicals exerted by whole extracts, proteins and phenolic compounds equally fed the reduction power. A more complex scenario was observed in the case of the capacity to inhibit hydroxyl radical formation, since other molecules, in addition to proteins and phenolic compounds, could be contributing, while the inhibition of lipid peroxidation was not correlated with the protein and phenolic contents. The enzymatic hydrolysis of extracts affected their antioxidant protection. Tandem mass spectrometry enabled eighteen different phenolic compounds to be identified. This technique also enabled observation of the presence of other molecules with antioxidant, anti-inflammatory, and neuroprotection activity (kynurenic acid, azelaic acid, sebaic acid, citric acid, and α,α-trehalose, and uridine) that could be playing important roles in the inhibition of hydroxyl radical formation and lipid peroxidation. Overall, the simultaneous presence of proteins, peptides, phenolic compounds, and other antioxidant molecules in an extract from apricot seeds led to a multifunctional and collaborative protection against oxidation that could not be exerted by extracts containing individual molecules.

## Figures and Tables

**Table 1:** Experiments established by Box–Behhken experimental design for the optimization of the percentage of EtOH, temperature, and time in PLE and protein content and TPC determined in the obtained extracts.

Experiments	EtOH (%)	Time (min)	Temperature (°C)	Protein Content (mg/100 mg Seeds)	Total Phenolic Content (mg GAE/100 mg Seeds)
1	50	15	105	12 ± 2	0.098 ± 0.004
2	50	25	170	14 ± 2	0.62 ± 0.01
3	0	15	170	22 ± 1	0.63 ± 0.04
4	100	25	105	10 ± 1	0.00 ± 0.01
5	50	15	105	11 ± 1	0.13 ± 0.02
6	0	5	105	13 ± 2	0.121 ± 0.003
7	100	15	40	10 ± 1	0.011 ± 0.004
8	50	5	170	15 ± 1	0.43 ± 0.01
9	50	15	105	11 ± 2	0.094 ± 0.001
10	50	15	105	10 ± 1	0.093 ± 0.002
11	0	25	105	12 ± 1	0.152 ± 0.002
12	100	15	170	10.9 ± 0.2	0.32 ± 0.01
13	0	15	40	14 ± 1	0.21 ± 0.01
14	50	25	40	9.4 ± 0.3	0.085 ± 0.001
15	50	15	105	11 ± 2	0.105 ± 0.004
16	100	5	105	9 ± 1	0.00 ± 0.01
17	50	5	40	8.8 ± 0.3	0.059 ± 0.002

**Table 2:** Conditions predicted by Box–Behnken experimental design for obtaining the highest protein yield, TPC, scavenging of ABTS and DPPH radicals, inhibition of hydroxyl radical formation, reducing power, and inhibition of lipid peroxidation by PLE and protein content and TPC determined in extracts.

	EtOH (%)	Time (min)	Temperature (°C)	Proteins Content (mg/100 mg Seed)	Total Phenolic Content (mg GAE/100 mg Seed)
Protein	0	14	170	20 ± 1 ^abc^	0.60 ± 0.02 ^b^
TPC	1.1	25	170	20 ± 1 ^bcd^	0.62 ± 0.01 ^bc^
Scavenging of ABTS radicals	0.25	25	170	20.3 ± 0.3 ^bcd^	0.67 ± 0.02 ^d^
Scavenging of DPPH radicals	7.4	25	170	19 ± 1 ^a^	0.64 ± 0.02 ^cd^
Inhibit of hydroxyl radical formation	0	5	170	21 ± 1 ^d^	0.45 ± 0.01 ^a^
Reducing power	0	24	170	21 ± 1 ^cd^	0.75 ± 0.01 ^e^
Inhibition of lipid peroxidation	76.4	15	170	19.3 ± 0.3 ^ab^	0.72 ± 0.02 ^e^

Different letters means statistical differences.

**Table 3:** Peptide content in the initial extracts and in the hydrolyzates obtained by gastrointestinal digestion (GD) or Alcalase hydrolysis.

	Peptide Content (mg/mL)		
Experiment	Initial Extract	Hydrolysate Obtained by GD	Hydrolysate Obtained by Alcalase Digestion
1	0.42 ± 0.07	0.65 ± 0.06	0.44 ± 0.05
2	0.6 ± 0.2	1.48 ± 0.07	1.1 ± 0.1
3	1.2 ± 0.2	3.6 ± 0.3	3.2 ± 0.2
4	0.07 ± 0.08	0.29 ± 0.08	0.18 ± 0.05
5	0.4 ± 0.1	0.6 ± 0.2	0.49 ± 0.06
6	0.92 ± 0.08	1.1 ± 0.1	1.4 ± 0.1
7	0.28 ± 0.04	0.1 ± 0.1	0.1 ± 0.1
8	0.6 ± 0.2	1.8 ± 0.2	0.8 ± 0.1
9	0.25 ± 0.07	0.7 ± 0.2	0.34 ± 0.04
10	0.25 ± 0.07	0.59 ± 0.05	0.36 ± 0.03
11	1.1 ± 0.1	1.7 ± 0.2	1.2 ± 0.2
12	0.23 ± 0.04	0.44 ± 0.05	0.29 ± 0.07
13	4.2 ± 0.2	5.3 ± 0.5	7.4 ± 0.7
14	0.21 ± 0.04	0.3 ± 0.1	0.29 ± 0.02
15	0.3 ± 0.1	0.4 ± 0.1	0.40 ± 0.05
16	0.13 ± 0.03	0.19 ± 0.03	0.16 ± 0.04
17	0.2 ± 0.1	0.30 ± 0.06	0.32 ± 0.08

**Table 4:** Phenolic compounds identified in extracts obtained under optimal conditions for the highest total phenolic content (TPC, 1.1% EtOH) and the inhibition of lipid peroxidation (ILP, 76.4% EtOH).

Compound	Fomula	Time (min)	[M-H]^−^ (*m/z*)	Fragments	Error (ppm)	Extract
Epigallocatechin	C15 H14 O7	2.09	305.06680	167/137/125/109	0.51	TPC
Caffeic acid	C9 H8 O4	3.48	179.03407	179/161/135/59	4.88	TPC
Neochlorogenic acid	C16 H18 O9	5.61	353.08826	191/179/135	1.10/0.07	TPC/ILP
Coumaroylquinic acid	C16 H18 O8	9.19	337.09305	163/119	1.01/0.38	TPC/ILP
(+)-Procyanidin B2	C30 H26 O12	11.44	577.13593	407/289/125	0.92/1.24	TPC/ILP
Catechin	C15 H14 O6	11.70	289.07196	245/203/125/109	0.58/0.37	TPC/ILP
3,4-Dihydroxyphenylpropionic acid	C9 H10 O4	12.23	181.04974	181/137/63	4.96	TPC
Feruloylquinic acid isomer	C17 H20 O9	12.46	367.10349	193/134	0.25/0.58	TPC/ILP
Chlorogenic acid	C16 H18 O9	12.56	353.08798	191/161	0.50/0.07	TPC/ILP
5-p-coumaroylquinic acid	C16 H18 O8	17.39	337.09351	191/173/163/93	1.19/0.92	TPC/ILP
Epichatechin	C15 H14 O6	18.01	289.07199	245/203/125/109	0.79/0.37	TPC/ILP
3′-O-methylcatechin	C16 H16 O6	18.38/18.29	303.087591	137/125	0.33/0.17	TPC/ILP
5-feruloylquinic acid	C17 H20 O9	20.27	367.10370	193/191/173/93	0.58/0.16	TPC/ILP
Ferulic acid	C10 H10 O4	21.48	193.04984	178/134	4.11/4.03	TPC/ILP
Phloretin	C15 H14 O5	22.53	273.07703	255/189/97	0.77	ILP
Quercetin	C15 H10 O7	23.97	301.03555	245/151	0.18	ILP
DL-4-hydroxyphenyllactic acid	C9 H10 O4	25.73	181.04970	153/121/109/59	4.96	TPC
Ethyl caffeate	C11 H12 O4	37.76	207.06563	179/161/135	3.36	ILP

## Data Availability

Data is contained within the article and in supplementary material.

## Supplementary Materials

The following supporting information can be downloaded at https://www.mdpi.com/article/10.3390/antiox11122354/s1: Figure S1: Images of extracts obtained by PLE under conditions established by Box–Benhken experimental design; Figure S2: Capacity of extracts to reduce the proliferation of HeLa cells; Figure S3: SDS-PAGE (A) and isoelectrophoretic (B) separation of proteins in extracts obtained under optimal conditions to obtain the highest protein yield, TPC, scavenging of ABTS and DPPH radicals, inhibition of hydroxyl radicals, reduction capacity, inhibition of lipid peroxidation, and reduction of oxidative damage in cells. Table S1: Additional compounds identified in extracts obtained under optimal conditions for the highest total phenolic content (TPC, 1.1% EtOH) and inhibition of lipid peroxidation (ILP, 76.4% EtOH); Table S2: Unknown compounds observed in extracts obtained under optimal conditions for the highest total phenolic content (TPC, 1.1% EtOH) and inhibition of lipid peroxidation (ILP, 76.4% EtOH).
