# Supplementary material for: Multifunctional and Collaborative Protection of Proteins, Peptides, Phenolic Compounds, and Other Molecules against Oxidation in Apricot Seeds Extracts
Source: Antioxidants (Basel). 2022 Nov 28;11(12):2354. doi: 10.3390/antiox11122354 (PMC9774911; doi:10.3390/antiox11122354)
Supplement: Supplementary file 1 [file antioxidants-11-02354-s001.zip › antioxidants-2043487-supplementary.pdf]

SUPPLEMENTARY MATERIAL

Multifunctional and Collaborative Protection Of Proteins, Peptides, Phenolics Compounds, and Other Molecules against Oxidation in Apricot Seeds Extracts

María Concepción García [1,2,*], Víctor Lombardo-Cristina [1], María Luisa Marina [1,2]

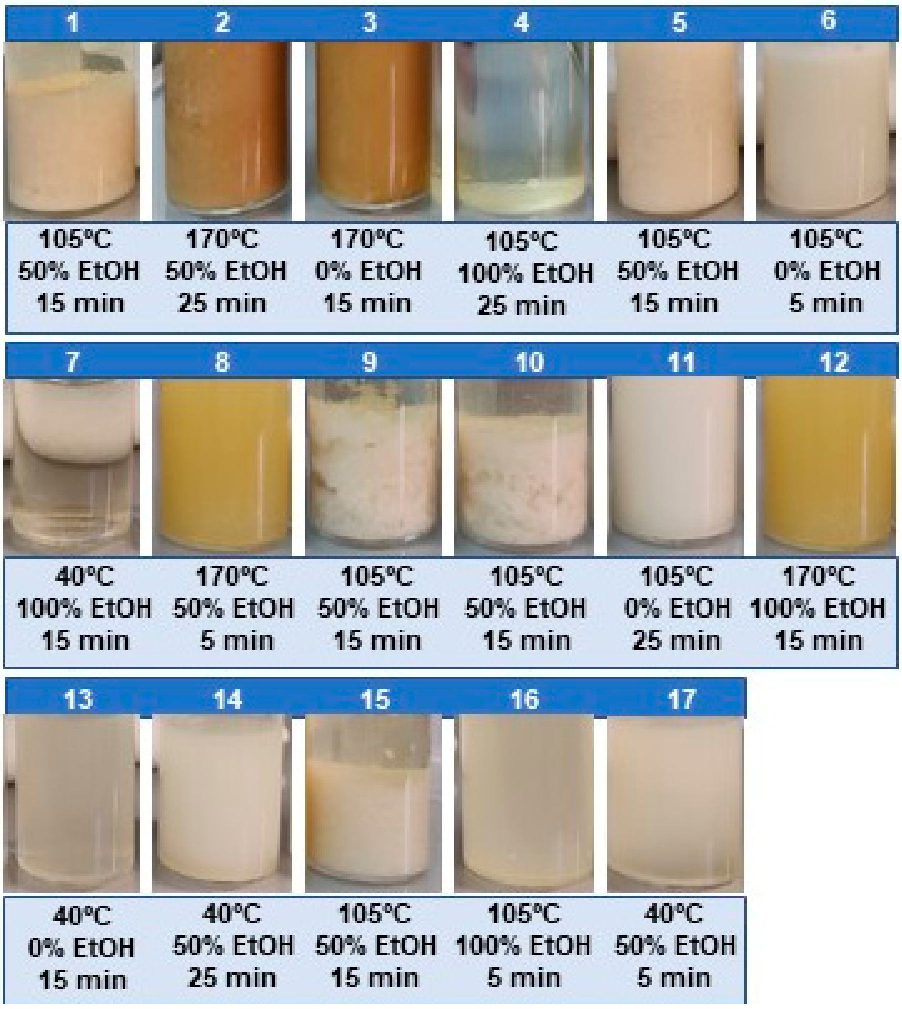

Figure S1. Images of extracts obtained by PLE under conditions established by Box-Benhken experimental design.

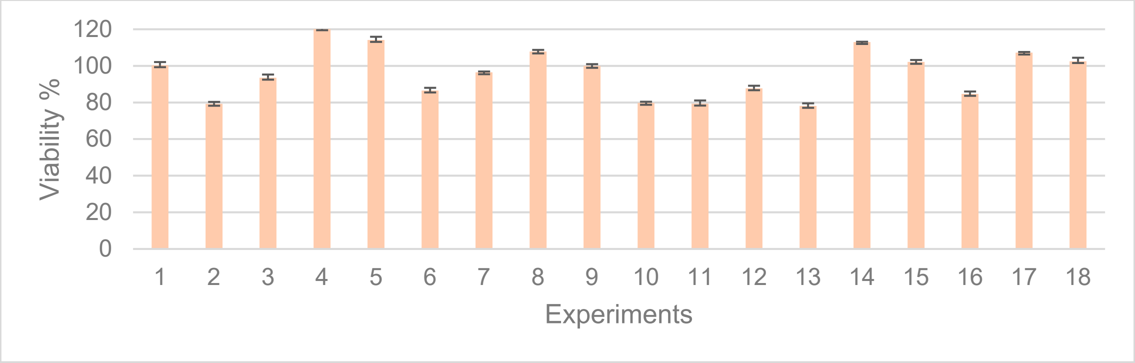

Figure S2. Capacity of extracts to reduce the proliferation of HeLa cells.

A)

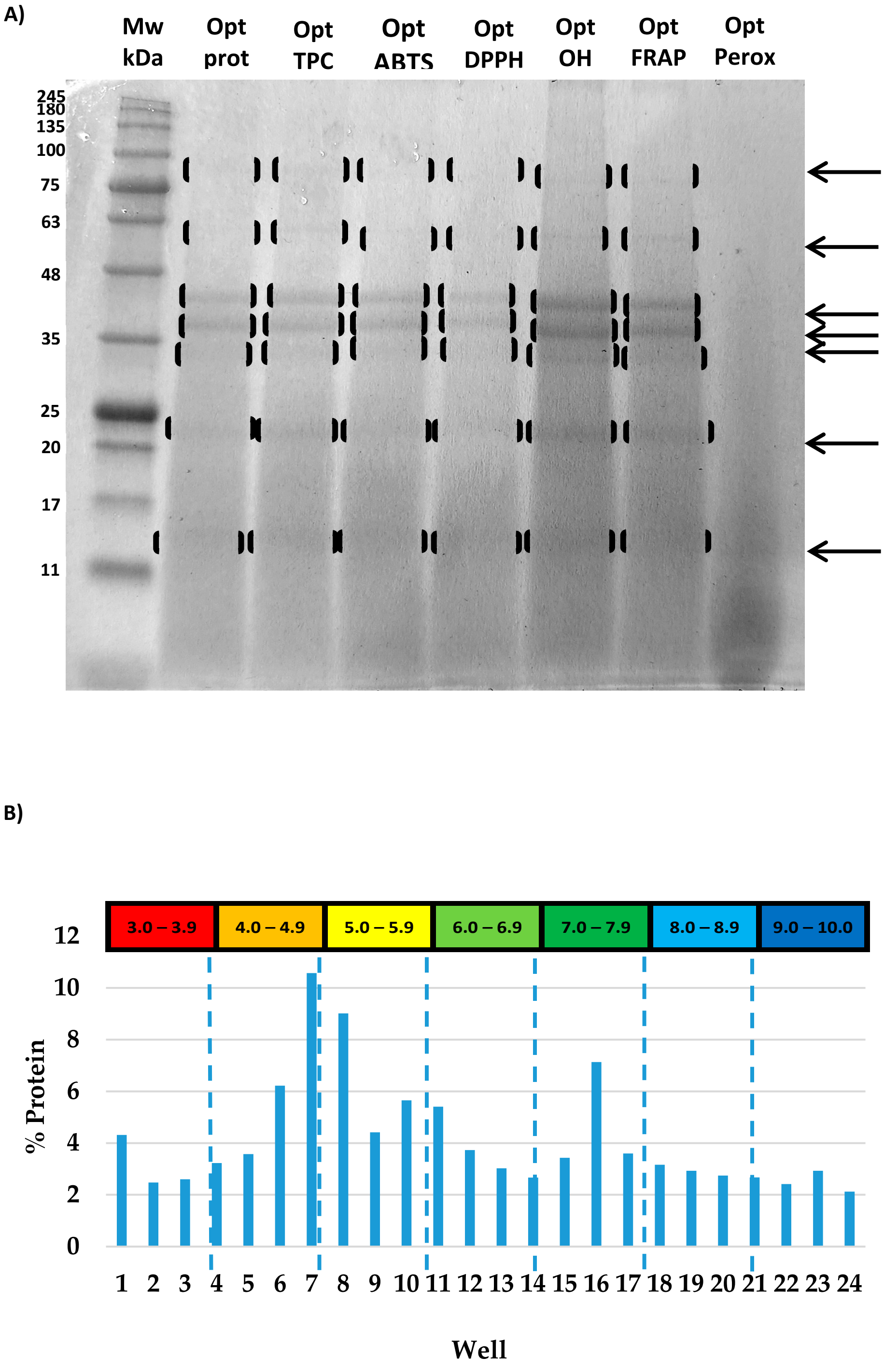

Figure S3. SDS-PAGE (A) and isoelectrophoretic (B) separation of proteins in extracts obtained under optimal conditions to obtain the highest protein yield, TPC, scavenging of ABTS and DPPH radicals, inhibition of hydroxyl radicals, reduction capacity, inhibition of lipids peroxidation, and reduction of oxidative damage in cells.

Table S1. Additional compounds identified in extracts obtained under optimal conditions for the highest total phenolic content (TPC, 1.1% EtOH) and inhibition of lipid peroxidation (ILP, 76.4% EtOH).

Compound	Fomula	RT (min)	$[M-H]^-$ (*m/z*)	Fragments	Error (ppm)	Extract
Sibiricose A3	C19 H26 O13	2.84	461.13031	137/93	0.53	TPC/ILP
DL-Tryptophan	C11 H12 N2 O2	4.84	203.08188	159/142/116/74	3.69	TPC/ILP
Guanosine	C10 H13 N5 O5	1.76	282.08426	150	0.68	TPC/ILP
Kynurenic acid	C10 H7 N O3	4.09	188.03441	144	4.64	TPC/ILP
Azelaic acid	C9 H16 O4	29.28	187.0967	125	4.66	TPC/ILP
Sebaic acid	C10 H18 O4	39.26	201.11252	201/183/139	3.53	TPC/ILP
Porphobilinogen	C10 H14 N2 O4	3.00	225.08751	181/165/153/112/82	2.59	TPC/ILP
α,α-Trehalose	C12 H22 O11	1.10	341.10867	113/101/89/59	4.05	ILP
Citric acid	C6 H8 O7	1.08	191.01883	111/88/87/57	4.67	TPC/ILP
Uridine	C9 H12 N2 O6	1.24	243.06206	199/182/152/110	0.83	TPC
N-acetyl-L-phenylalanine	C11 H13 N O3	12.95	206.08156	164/58	3.64	TPC

Table S2. Unknown compounds observed in extracts obtained under optimal conditions for the highest total phenolic content (TPC, 1.1% EtOH) and inhibition of lipid peroxidation (ILP, 76.4% EtOH).

Fomula	RT (min)	$[M-H]^-$ (*m/z*)	Fragments	Error (ppm)	Extract
C6 H12 O7	1.33	195.0500	195/159/129/97/75	0.78	TPC/ILP
C15 H12 O6	2.5	287.05634	269/161/125	0.35	TPC/ILP
C17 H20 O6	10.78/10.58	319.11908	271/256/241	0.86/0.67	TPC/ILP
C15 H10 O6	9.31	285.10928	128	0.23	ILP
